# Molecular Structure-Based Screening of the Constituents of *Calotropis procera* Identifies Potential Inhibitors of Diabetes Mellitus Target Alpha Glucosidase

**DOI:** 10.3390/cimb44020064

**Published:** 2022-02-21

**Authors:** Cynthia A. Adinortey, Gabriel B. Kwarko, Russell Koranteng, Daniel Boison, Issaka Obuaba, Michael D. Wilson, Samuel K. Kwofie

**Affiliations:** 1 Department of Molecular Biology and Biotechnology, School of Biological Sciences, University of Cape Coast, Cape Coast CC 033, Ghana; cadinortey@ucc.edu.gh; 2 West African Centre for Cell Biology of Infectious Pathogens, Department of Biochemistry, Cell and Molecular Biology, College of Basic and Applied Sciences, University of Ghana, Legon, Accra LG 54, Ghana; kwarkogabriel@gmail.com; 3 Department of Biomedical Engineering, School of Engineering Sciences, College of Basic & Applied Sciences, University of Ghana, Legon, Accra LG 77, Ghana; russct27@gmail.com; 4 Department of Biochemistry, School of Biological Sciences, University of Cape Coast, Cape Coast CC 033, Ghana; daniel.boison@ucc.edu.gh (D.B.); iobuaba@gmail.com (I.O.); 5 Department of Parasitology, Noguchi Memorial Institute for Medical Research (NMIMR), College of Health Sciences (CHS), University of Ghana, Legon, Accra LG 581, Ghana; Mwilson@noguchi.ug.edu.gh

**Keywords:** diabetes, *Calotropis procera*, alpha glucosidase, cheminformatics, molecular docking, molecular dynamics simulations

## Abstract

Diabetes mellitus is a disorder characterized by higher levels of blood glucose due to impaired insulin mechanisms. Alpha glucosidase is a critical drug target implicated in the mechanisms of diabetes mellitus and its inhibition controls hyperglycemia. Since the existing standard synthetic drugs have therapeutic limitations, it is imperative to identify new potent inhibitors of natural product origin which may slow carbohydrate digestion and absorption via alpha glucosidase. Since plant extracts from *Calotropis procera* have been extensively used in the treatment of diabetes mellitus, the present study used molecular docking and dynamics simulation techniques to screen its constituents against the receptor alpha glucosidase. Taraxasterol, syriogenin, isorhamnetin-3-O-robinobioside and calotoxin were identified as potential novel lead compounds with plausible binding energies of −40.2, −35.1, −34.3 and −34.3 kJ/mol against alpha glucosidase, respectively. The residues Trp^481^, Asp^518^, Leu^677^, Leu^678^ and Leu^680^ were identified as critical for binding and the compounds were predicted as alpha glucosidase inhibitors. Structurally similar compounds with Tanimoto coefficients greater than 0.7 were reported experimentally to be inhibitors of alpha glucosidase or antidiabetic. The structures of the molecules may serve as templates for the design of novel inhibitors and warrant in vitro assaying to corroborate their antidiabetic potential.

## 1. Introduction

Diabetes mellitus (DM) is a disorder characterized by persistent elevated levels of blood glucose, consequences of impaired insulin production, resistance or both [1,2]. The disorder when uncontrolled is associated with chronic complications, which include damage to the eyes, kidney and the cardiovascular system. Although DM is classified into four main categories, the two pervasive ones are type-1-diabetes mellitus (T1DM) and type-2-diabetes mellitus (T2DM). T1DM is caused by absolute lack of insulin whereas T2DM is mainly due to ineffective insulin action often referred to as insulin resistance due to defective insulin secretion or irresponsiveness on the part of insulin receptors. Over 90% of DM cases worldwide are T2DM [3]. Several driving factors of T2DM globally include overweight, obesity, sedentary lifestyles, and consumption of red and processed meat as well as sugar-sweetened beverages.

The disease burden of DM and its complications poses a major global health threat. The disorder affects approximately 10% of the world’s population [2,4]. The International Diabetes Federation (IDF) estimated that of adults between the ages of 20 and 79 years, 415 million had DM globally in 2015 and this prevalence is expected to increase to 629 million by 2045 [1,2,3,5]. In 2013, the Global Burden of Disease Study identified DM as the ninth major cause of reduced life expectancy [3]. DM and its complications have contributed enormously to the burden of mortality and disability worldwide.

The activities of key enzymes including alpha glucosidase have been associated with DM mechanisms [6,7,8]. Alpha glucosidase is present in the brush border membrane of the intestines. It is a calcium-containing enzyme that hydrolyzes starch and disaccharides into glucose [9]. It catalyzes the hydrolysis of α-(1,4)-glycosidic linkage of sugar, releasing free monosaccharide (α-D-glucose) during the final step of glucose digestion [8]. This enzyme plays a crucial role in DM and has been a target for managing diabetes mellitus. Compounds capable of inhibiting alpha glucosidase enzyme can slow carbohydrate digestion and absorption. Thus, independent of insulin, they can control the peaks of meal-related hyperglycemia [9]. Alpha glucosidase inhibitors (AGIs) are a unique class of antidiabetic drugs approved for the prevention and management of T2DM [10]. Currently, three types of AGIs (miglitol, acarbose and voglibose) are available for treatment of T2DM, of which acarbose is the most widely used [11].

Though several synthetic AGIs being studied exhibit good efficacy, many have been withdrawn from clinical trials because of relatively low efficacy and several adverse effects [12]. Notwithstanding the considerable progress made in the discovery of oral anti-hyperglycemic agents, the search for new drugs continues because the existing standard drugs have several deficiencies ranging from limited efficacy to enormous side effects, such as weight gain, poor pharmacokinetics and chronic tissue damage [13]. A recent paradigm shift is the study of natural products from medicinal plants as antidiabetic agents [14,15,16]. Natural products and their derivatives have been recognized as sources of structurally diverse therapeutic agents. These studies have employed the use of extracts from plants, such as *Calotropis procera*, *Calotropis gigantea*, *Hyophorbe lagenicaulis*, *Lepidium sativum*, *Ocimum campechianum*, *Psiadia punctulata* and *Ervatamia microphylla*, as inhibitors of key enzymes involved in diabetes mellitus, such as alpha glucosidase, aldose reductase and alpha amylase [17,18]. 

*Calotropis procera* is a small popular plant found in tropical and sub-tropical regions of the world. It is widely used in traditional medicinal systems in countries such as India, Saudi Arabia, Sudan, Nigeria and Ghana [17,19,20,21,22]. The different parts of the plant contain many biologically active chemical groups including cardenolides, steroids, tannins, glycosides, phenols, terpenoids, sugars, flavonoids, alkaloids and saponins. In addition to its antidiabetic effects, it is reported to have anticancer, antibacterial, antioxidant and antidiarrheal effects. *In vitro* experiments conducted on streptozotocin-induced diabetic rats using leaf extracts showed strong inhibition of alpha glucosidase [17,18,23,24,25]. Therefore, compounds from extracts from different parts of the plant can be explored for their antidiabetic effects. A review article on the constituents and pharmacological properties of *Calotropis procera* provided insight into some isolated compounds from the plant [19]. Compounds such as calotropin, calotropagenin, isorhamnetin-3-O-rutinoside, calotoxin, calactin, uscharin, and others are reported to be present in different parts of the plant [19]. Several in silico studies identified inhibitors from a plethora of natural products against key receptors in the disease mechanisms including alpha glucosidase [6,8,16,26,27,28,29]. Since inhibition of these enzymes is directly associated with treatment of DM [30,31,32,33,34,35], identifying potentially new AGIs from plants is an essential basis for drug discovery. Therefore, this work aims to identify natural products derived from the *Calotropis procera* plant as potential inhibitors of the drug target alpha glucosidase via a plethora of molecular informatics techniques. The work also seeks to predict the pharmacological profiles and gain novel insights into the mechanisms of binding, as well as predicting the biological activity to augment the search for potential lead compounds. 

## 2. Materials and Methods

### 2.1. Preprocessing of Target Structure

The 3D structure of human lysosomal alpha glucosidase was obtained from the Protein Data Bank (https://www.rcsb.org; accessed on 17 April 2021) [36] with PDB ID of 5NN8 and a resolution of 2.45 Å [37]. PyMol [38] was used to visualize the target structure. All water molecules and co-ligands were removed and the final molecule saved in a (.pdb) file. GROMACS 2018 was used to perform molecular dynamics simulations by converting the (.pdb) file of 5NN8 to a (.gro) compatible file [39,40]. By using the steepest descent algorithm and GROMOS96 force field, the target structure was minimized and equilibrated at a temperature of 300 K and constant pressure [41]. The final (.gro) file was converted back to (.pdb) file for molecular docking.

### 2.2. Molecular Docking of Compounds against Alpha Glucosidase

A total of 32 compounds (Table 1), known to be from different parts of the *Calotropis procera* plant were curated together with 25 known inhibitors of alpha glucosidase (Supplementary Table S1). The ligands retrieved from the database (https://pubchem.ncbi.nlm.nih.gov; accessed on 12 March 2021) [42] were energy minimized using OpenBabel environment via PyRx [43]. The energy minimization was done using the Universal Force Field (UFF) [44]. All ligands were converted to a (.pdbqt) formats. The target structure of 5NN8 underwent energy minimization. Known active site residues were selected within a grid box of dimensions X: 53.23 Å, Y: 64.63 Å and Z: 87.14 Å; and center X: 51.09 Å, Y: 50.41 Å and Z: 60.83 Å within the AutoDock Vina environment of PyRx. The resulting docked poses of the ligands based on cluster analysis within the protein target structure were visualized using PyMol and the resulting complexes were used for characterization of binding mechanism.

### 2.3. Mechanism of Binding Characterization

LigPlot^+^ (v1.4.5) [47,48] was used to characterize the binding mechanisms between the target protein structure and selected compounds based on their hydrogen bonds and hydrophobic interactions. Residues from the interactions of the selected compounds from the plant and that of known inhibitors with the target structure were analyzed to identify common residues that might possibly be critical in the binding of ligands.

### 2.4. Validation of Docking Protocol

LigAlign [49] was employed in the authentication of the docking protocol. The ligand, acarbose from the protein complex 5NN8 was removed from the co-crystalized complex and redocked. The resulting structure was saved as a (.pdb) file and uploaded into PyMol together with the solved complex (5NN8) from the Protein Data Bank. LigAlign was then used to calculate the deviation between the superimposed redocked and co-crystalized ligands. Ligplot^+^ was used to view similar hydrophobic and hydrogen bonding residues that overlapped during superimposition in both complexes.

### 2.5. Prediction of Biological Activity of Compounds

Prediction of activity spectra for substance (PASS) [50] was used for the prediction of the biological activity of selected compounds based on a training dataset of known substrates present in its database. Simplified molecular input line entry system (SMILES) of the compounds were used to predict the biological activity of the compounds with a focus on antidiabetic related biological activities.

### 2.6. Molecular Dynamics Simulations of Protein-Ligand Complexes

Complexes with optimum binding affinities were subjected to molecular dynamics simulation to observe the flexibility and stability of the complexes. The molecular dynamics simulation was performed with GROMACS 2018 [39]. PRODRG was used to generate the ligand topologies which were converted to complex (*.gro) files. Ligand-receptor complexes were solvated in a water dodecahedron box with an adjusted 1 Å distance and neutralized ions environment. The energy of the system was minimized using the steepest descent algorithm coupled with the GROMOS43A force field. Using periodic boundary conditions, the complex system underwent equilibration and the resulting system was used for running the molecular dynamics production for 50 ns.

### 2.7. MM-PBSA Calculations of Receptor-Ligand Complex

Molecular mechanics Poisson–Boltzmann surface area (MM-PBSA) calculations of the complexes were carried out using g_mmpbsa. Furthermore, g_mmpbsa was developed to enable the use of the MM-PBSA method in conjunction with the GROMACS package [51]. MM-PBSA calculates the binding free energy components and the discrete energy contributions of the residues. This is achieved primarily by using a thermodynamic path that includes solvation [52]. Graphs of the binding free energies were obtained with the R programming package [53].

### 2.8. Structural Exploration of Potential Leads

Structural similarity searches were done at a threshold of 0.7 via DrugBank 5.0 database to evaluate the potential diabetic activity and possible mechanisms of action from similar compounds.

## 3. Results and Discussion

Molecular informatics studies on natural product compounds contribute to our understanding of their pharmacological potentials. However, not many cheminformatics studies have been undertaken on compounds isolated from *Calotropis procera* plants for use as potential antidiabetics. This is what necessitated our study since various components of *Calotropis procera* have been shown to exhibit antidiabetic activity.

### 3.1. Preprocessing of Alpha Glucosidase as a Target Structure

The target structure of human alpha glucosidase used for analysis was retrieved from the Protein Databank (PDB ID: 5NN8) with a resolution of 2.45 Å. The structure is an asymmetric monomer comprised of an N-terminal trefoil type-p domain followed by a β-sheet domain, catalytic (β/α), proximal and distal β-sheet domains at the C-terminus [37]. In addition, it has glycan structures of various lengths with five of them in the crystal structure notably at Asn^140^, Asn^233^, Asn^390^, Asn^470^ and Asn^652^ usually used for glycosylation. Glycosylation plays a critical role in determining protein structure, function and stability [54].

The active site appears in the catalytic domain with residues Pro^125^, Asp^282^, Trp^376^, Asp^404^, Ile^441^, Trp^481^, Asp^518^, Met^519^, Arg^600^, Trp^613^, Asp^616^, Trp^618^, Phe^649^, His^674^, Gly^896^ and Glu^945^. The active site is characterized by numerous residues that span between narrow sub-sites. In the active site are two residues, Asp^518^ and Asp^616^, which are catalytic residues critical for the hydrolysis of glycosidic linkage in sugars [37]. In addition to the active site, there are residues reported to be associated with a secondary substrate binding site near the C-terminal ends of the β strands of the catalytic (β/α) and these residues are Asp^91^, Ala^93^, Gly^123^, Gln^124^, Trp^126^, Cys^127^, His^432^, Arg^437^, Gly^434^, Gly^435^, His^742^, Leu^756^ and Gln^757^. Enzymes of the glycosyl hydrolase (GH31) family exhibit similar features, that is, an active site and secondary substrate binding site as revealed by studies conducted with different enzymes [28,55].

Biomolecules such as proteins exist in a dynamic state of motion with reasonably high energy and instability [56]. Proteins work best when their energies are minimized. It was, therefore, appropriate to ensure that the target structure was well equilibrated with a minimized energy for molecular docking studies. This was achieved by using GROMACS to perform the initial molecular dynamics of the target structure subjected to the GROMOS96 force field with an aftermath root mean square deviation (RMSD) of 0.25 Å (Supplementary Figure S1).

### 3.2. Molecular Docking against Alpha Glucosidase as a Target Structure

All ligands used for molecular docking were obtained from literature (Table 1). This was also achieved by exhaustively identifying isolated compounds extracted not only from the leaves but also from other parts of the *Calotropis procera* plant [18,19,20,21,22,25,57]. The majority of the compounds used in this study were isolated from the stem, latex and the leaf, while the rest were isolated from the root and root bark. Earlier reports cited have stated that compounds isolated from the leaves (dried or fresh) have been shown to exhibit antidiabetic effects in vitro. However, it appears there is scanty data on the antidiabetic effects of compounds isolated from other parts of the plant, hence the purpose of this work was to investigate the antidiabetic effects through in silico approach.

In view of the effects of alpha glucosidase on postprandial glucose, there have been studies with both natural and synthetic compounds that act as AGIs through in silico and in vitro approaches. Some of these inhibitors, including Food and Drug Authority (FDA) approved drugs acarbose and miglitol, were curated with their corresponding IC_50_ values (Supplementary Table S1) [10,58,59,60,61,62,63]. Acacetin, hesperitin-5-O-glucoside, plicatanoside and some other compounds from natural products have been reported to be alpha glucosidase inhibitors through in vitro studies [30,55,64,65].

**Figure 1:**
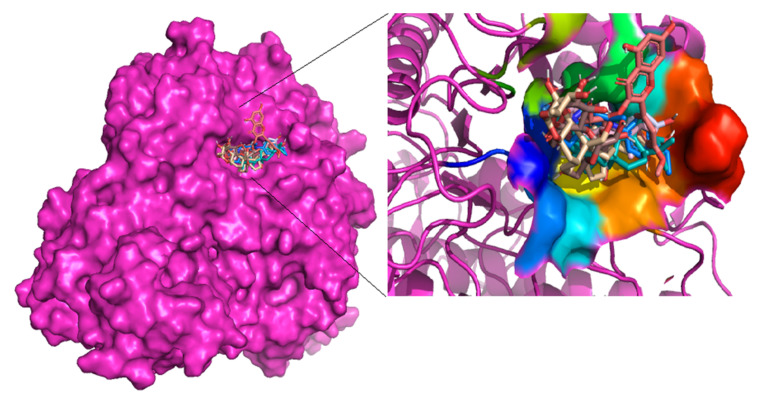
Schematic representation of constituent compounds of *Calotropis procera* docked at the active site of alpha glucosidase.

A single grid box for molecular docking was set to cover residues in the reported active site and the putative secondary substrate binding sites [37]. PyMol provided a productive environment to explore the protein-ligand complex to identify whether the ligands were firmly docked in the active site. Figure 1 shows cartoon representations of almost all the compounds docked deep inside the binding site of the alpha glucosidase. The best poses for all the compounds were selected based on cluster analysis of docking results. Cluster analysis gives information on the binding position having the highest probability with respect to the stability of the protein-ligand complex [66].

### 3.3. Comparison of Binding Energies of Selected Compounds of Calotropis procera and Known Inhibitors

The binding affinity of a ligand to a protein is the strength of the binding interactions between the biomolecules [67]. Table 2 provides details on the binding energies of the selected compounds from *Calotropis procera* and the known inhibitors of alpha glucosidase (Supplementary Table S2). The low binding energies give an indication of a better binding affinity between the ligands and the target structure of alpha glucosidase [67]. The process of molecular recognition constitutes the basis of all processes in living organisms [68].

Taraxasterol had the lowest binding energy of −40.2 kJ/mol whilst thioacetic acid exhibited the highest binding energy of −10.9 kJ/mol. Apigenin-7-O-rutinoside exhibited the lowest binding energy of −38.1 kJ/mol compared to 4-(p-toluenesulfonamide)-3,4-dihydroxy chalcone which exhibited the highest binding energy of –23.4 kJ/mol amongst the known inhibitors (Supplementary Table S2). The three FDA approved drugs acarbose, voglibose and miglitol had binding energies of −34.3, −24.7 and −23.4 kJ/mol, respectively. Aside from Apigenin-7-O-rutinoside, some of the known inhibitors such as plicatanoside and rutin both showed high binding affinity with binding energy value of −37.6 kJ/mol. Experimental work on these two compounds showed respective IC_50_ values of 111.23 ± 0.65 µM [69] and 173.58 ± 1.23 µM [27]. This is much lower than IC_50_ of miglitol, which has been experimented to have a value of 465.1 µM [60].

In other studies, acarbose on the other hand showed lower IC_50_ values of 0.59 ± 0.14 µM [70] and 996 nM [55], although having a higher binding energy of −34.3 kJ/mol than plicatanoside and rutin in this molecular docking study. Several studies have used the binding affinity of acarbose as a positive control to prefilter the existing docking library for downstream analysis [55,71,72,73]. To proceed with plausible leads for downstream analysis, we first eliminated compounds of *Calotropis procera* that have been already experimentally shown to possess activity against alpha glucosidase. This was to make sure that final leads were novel. The following 10 compounds, namely, β-amyrin [74], ursolic acid [74,75], luteolin [65,67,76,77], isorhamnetin-3-O-rutinoside [27], quercetin-3-rutinoside [78], kaempferol-7-0-glucoside, apigenin-7-0-glucoside [27], glibenclamide [79], β-sitosterol [76] and α-amyrin [80] have been proven to show good activity against alpha glucosidase. In addition to this, further elimination considered acarbose as our control compound, thus compounds with binding energy greater than −34.1 kJ/mol were also eliminated. This narrowed the available compounds down to a total of nine. 

Therefore, isolated compounds from the *Calotropis procera* that exhibited better binding energy values which are comparable to that of acarbose included taraxasterol (−40.2 kJ/mol), voruscharin (−39.3 kJ/mol), 3-epimoretenol (−36.8 kJ/mol), lactucerol (−36.4 kJ/mol), uscharin (−35.1 kJ/mol), syriogenin (−35.1 kJ/mol), benzoyllineolone (−34.7 kJ/mol), isorhamnetin-3-O-robinobioside (−34.3 kJ/mol) and calotoxin (−34.3 kJ/mol). Molecular docking with the known inhibitors served as a guide for the selection of compounds based on binding energies. The range of binding energies of these known AGIs were comparable to those of the shortlisted isolated compounds from *Calotropis procera*, thereby serving as a benchmark. As such, compounds isolated from plants with energies below −34.3 kJ/mol could be investigated as potential AGIs [27,29,60,62,81,82].

### 3.4. Molecular Interactions with Alpha Glucosidase

Molecular interactions studies are vital for understanding the mechanism of biological regulations and they provide a theoretical basis for the design and discovery of new drug targets [83,84]. This makes protein-ligand interactions a prerequisite for signal transduction, immunoreaction and gene regulation [83]. An understanding of the protein-ligand interactions is therefore central to understanding biology at the molecular level [81,85,86]. Weak intermolecular attractions such as hydrogen bonding and optimized hydrophobic interactions both stabilize ligands at the active sites and can alter the binding affinity and efficacy [87]. Hydrogen bond distance gives an indication of the strength of the hydrogen bond and a strong hydrogen bond is observed when hydrogen bond distance is below 3 Å and angles greater than 150° [88]. A total of seven out of the shortlisted nine compounds formed hydrogen bonds with the receptor. Nonetheless, hydrophobic interactions were present for all the nine compounds in complex with the receptor (Table 2).

**Figure 2:**
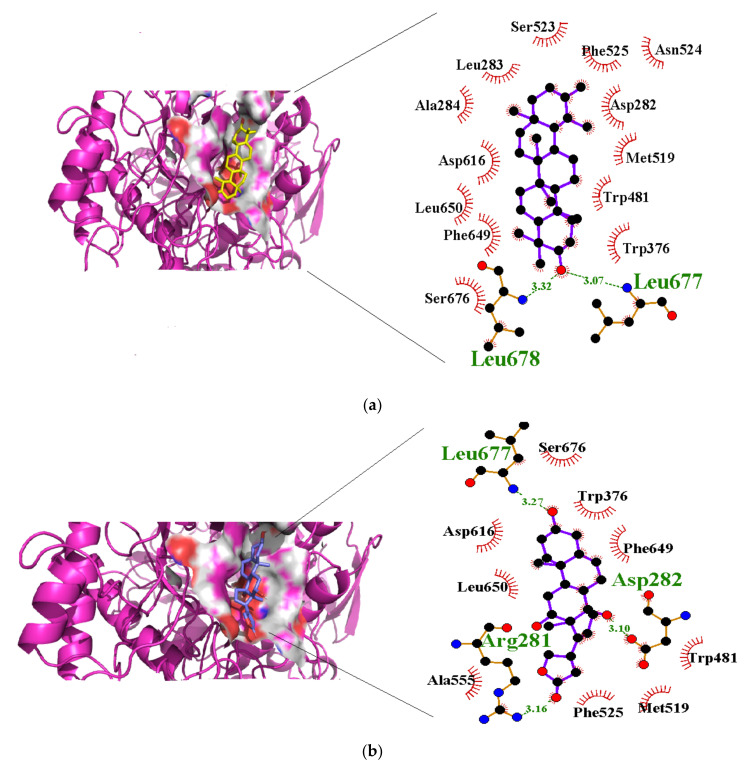

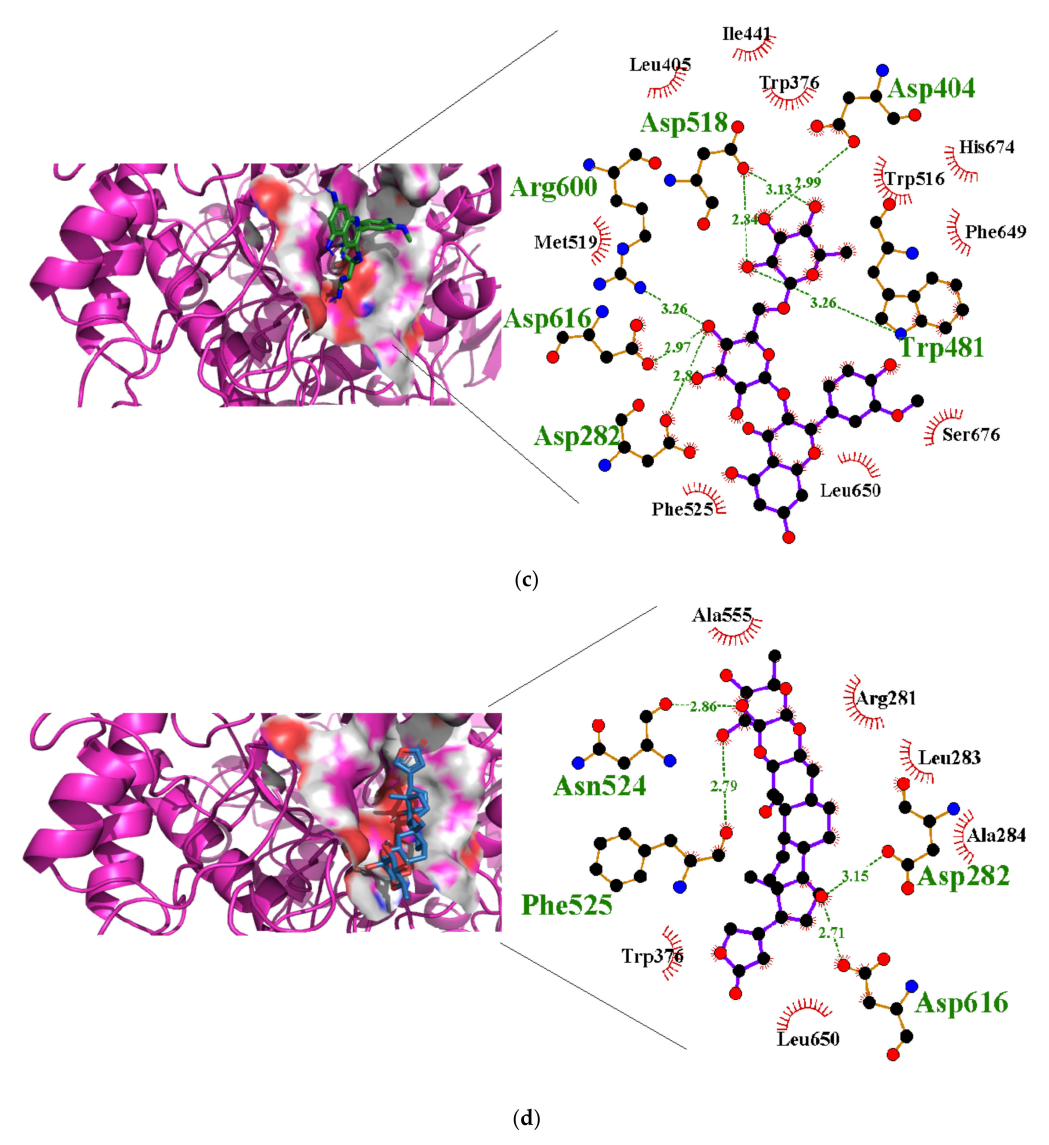
Schematic view and Ligplot^+^ representation of mechanism of interactions of (**a**) taraxasterol; (**b**) syriogenin (**c**) isorhamnetin-3-O-robinobioside; and (**d**) calotoxin in the active site of alpha glucosidase. Ligands are presented as purple-colored sticks surrounded by hydrophobic contacts in red arcs and hydrogen bonds in green dotted lines.

In a previous study on triazoloquinazolines as a new class of potent inhibitors, it was deduced that the number of hydrogen bonds formed between triazoloquinazolines and alpha glucosidase was an indication of higher activity [89]. Hydrogen bonds form the major binding interaction modes between compounds and active site residues which maintain complex stability. Therefore, compounds with good binding affinities coupled with at least three hydrogen bonds have the tendency to exhibit alpha glucosidase inhibition [57,82,89]. Compounds such as 3-epimoretenol and lactucerol could be unfavorable with no hydrogen bond interactions. In addition, compounds such as uscharin and benzoyllineolone had only one hydrogen bond with residues Asp^616^ and Asp^282^, respectively. The compound with the highest binding affinity was taraxasterol (Figure 2a). This formed hydrogen bonds with two residues, Leu^677^ and Leu^678^, and bond lengths greater than 3 Å. Compounds with hydrogen bonds greater than or equal to three were explored. This included voruscharin, syriogenin, isorhamnentin-3-O-robinobioside and calotoxin. Voruscharin formed three hydrogen bonds with Arg^281^, Asp^616^ and Leu^678^. Asp^616^ possessed a shorter bond length of 2.36 Å. The other residues had bond lengths above 3 Å. Shorter bond lengths are much preferred since they strengthen bonding. Syriogenin formed four hydrogen bonds with active site residues Arg^281^, Asp^282^, Asp^616^ and Leu^677^ (Figure 2b). All the bond lengths exceeded the threshold of 3 Å except for Asp^616^, which had 2.88 Å. Isorhamnetin-3-O-robinobioside on the other hand formed seven hydrogen bonds with Asp^282^, Asp^404^, Trp^481^, Asp^518^, Arg^600^ and Asp^616^. Shorter bond lengths of 2.82 Å, 2.81 Å and 2.99 Å were attributed to these three residues Asp^282^, Asp^518^ and Asp^616^, respectively. The rest showed greater bond lengths above 3 Å. Calotoxin formed four hydrogen bonds with four residues Asp^282^, Asn^524^, Phe^525^ andAsp^616^. The bond length of Asp^282^ was 3.15 Å, which was above 3 Å. Relatively shorter bond lengths of 2.86 Å, 2.79 Å and 2.71 Å were formed by the last three respective residues in bonding with calotoxin mentioned previously. The shorter the length of the hydrogen bond with a correct angle of geometry, the greater the strength of the hydrogen bond and this influences the stability of a ligand in a protein structure [88]. Residues contribute to the stability of the ligands inside the protein by serving as hydrogen bond donors or acceptors [90]. Molecular interactions of acarbose with the protein also revealed 12 hydrogen bond interactions with seven residues Asp^282^, Asp^404^, Asn^524^, Phe^525^, Arg^600^, Asp^616^ and His^764^. All of its hydrogen bond lengths were below 3.0 Å except His^764^ with a bond length of 3.05 Å (Table 2). Inferring from the shorter bond length of acarbose, it could be suggested that its shorter bond lengths contribute to its stability and overall good inhibitory activity it possesses as an FDA approved drug. As such, isorhamnetin-3-robinobioside (Figure 2c) and calotoxin (Figure 2d) interacting with three residues each with bond lengths less than 3 Å can possibly possess a better advantage over the rest of the nine compounds.

### 3.5. Validation of Docking Protocol

**Figure 3:**
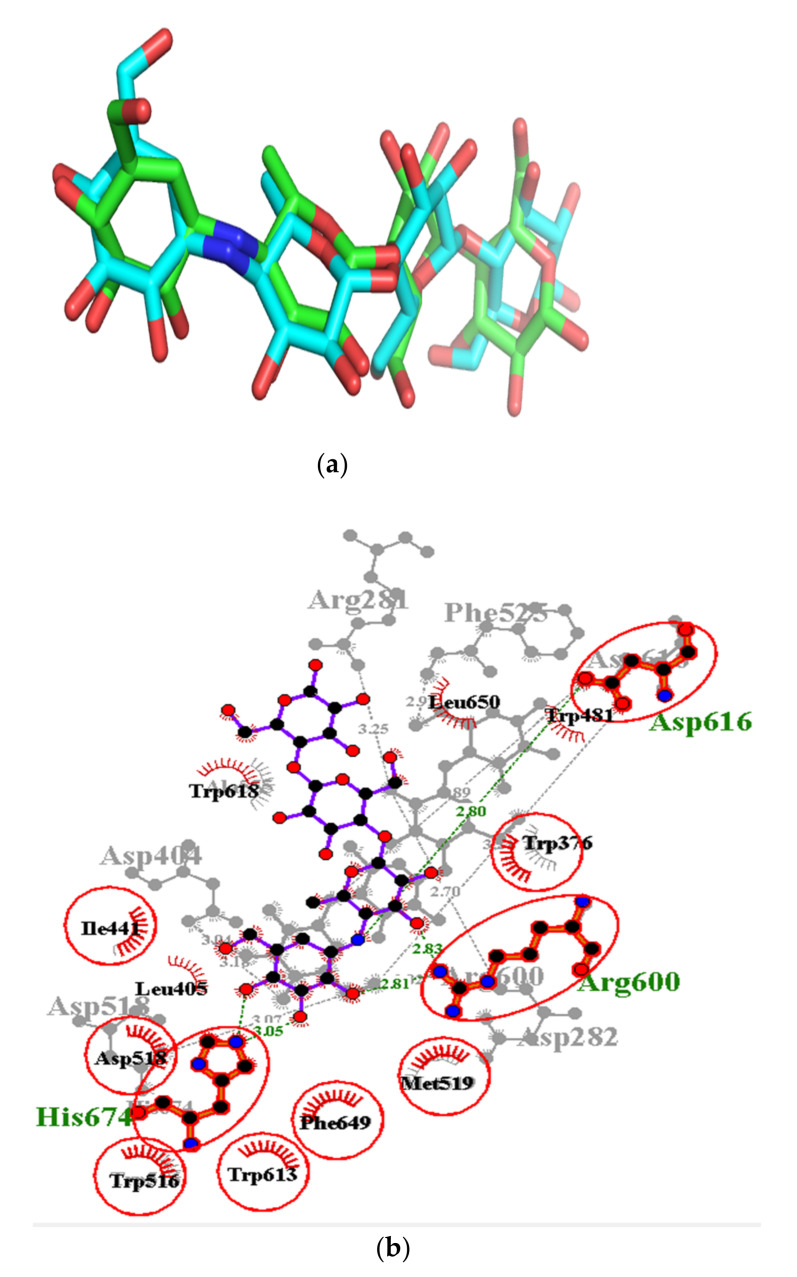
Docking validation via (**a**) superimposition of the redocked (green) onto the co-crystalized ligand acarbose (cyan) using LigAlign with computed RMSD of 2.206 Å, and (**b**) overlapping residues after superimposition of redocked and co-crystallized (5NN8) complexes. Identical critical hydrogen bonding residues (Arg^600^, Asp^616^ and His^674^) to 5NN8 are highlighted in red.

Validation of the docking protocol is essential in evaluating the efficiency by assessing the binding modes of the ligands in the active site region [49,91]. In our study, LigAlign script in PyMol environment was used in validating the protocol by the superimposition of ligands. This superimposes a redocked ligand pose onto a crystallographic pose and then computes the RMSDs. Figure 3 illustrates the superimposition of the redocked acarbose (green) onto the co-crystalized acarbose ligand (colored blue) with computed RMSD of 2.206 Å (Figure 3a), which was slightly above the threshold of 2.0 Å [92,93]. Nonetheless, redocked compound (acarbose) was able to simulate the binding of three critical hydrogen bonding residues (Arg^600^, Asp^616^ and His^674^) and seven hydrophobic bond residues (Trp^376^, Leu^405^, Ile^441^, Trp^516^, Asp^518^, Trp^613^ and Phe^649^) (Figure 3b), which have already been identified in the crystal structure in complex with acarbose. LigAlign uses ligand-based active site alignment and this technique is widely adopted for structural analysis of protein-ligand complexes [49].

### 3.6. Prediction of Antidiabetic Activity of Selected Compounds

The nine compounds with low binding energies were selected for biological activity predictions using PASS [50]. PASS uses a dataset of about 35,000 known active substrates to predict pharmacological effects and biochemical mechanisms based on the structural formula of a queried substance. This approach efficiently predicts new mechanisms of actions and biological activity [50,94,95]. Table 3 shows the results of the biological activities of predicted compounds using PASS with a focus on alpha glucosidase activity.

The PASS predictions were based on their probable activity (Pa) and probable inactivity (Pi). When Pa is greater than Pi (Pa > Pi), it is worth exploring the biological activity [50,95]. However, when the activity is confirmed experimentally, then the substance is a new chemical entity for the biological activity [50]. PASS prediction results of the selected compounds were collated together with their Pa and Pi values. Six out of the nine showed anti-glucosidase activity or at least some related antidiabetic activity (Table 3). Focusing on alpha glucosidase inhibition activity, isorhamnetin-3-O-robinobioside had the highest Pa of 0.808 with Pi of 0.001. Lactucerol showed a Pa of 0.200 with Pi of 0.050 and taraxasterol showed a Pa of 0.200 with Pi of 0.005. This was followed by syriogenin showing a Pi of 0.102 with Pa of 0.029 and finally calotoxin having a Pa of 0.101 and Pi of 0.029. Moreover, taraxasterol and 3-epimoretenol were predicted with related activity against some target proteins for diabetes. This included hydroxysteroid dehydrogenase, protein tyrosine phosphate and 17-beta-hydroxysterol dehydrogenase [96]. Voruscharin, usharin and benzoyllineolone showed no AG predicted activities and as a result were not analyzed further. Since the compounds 3-epimoretenol and lactucerol were observed to form no hydrogen bonds, they were not used for downstream analysis despite their predicted alpha glucosidase activity. Similarly, uscharin and benzoyllineolone formed only a single hydrogen bond.

The rest of the compounds, taraxasterols, syriogenin, isorhamnetin-3-O-robinobioside and calotoxin were found to have plausible binding energies, hydrogen bond interactions, bond lengths and predicted biological activities. These were considered for molecular dynamic simulations.

### 3.7. Molecular Dynamics of Protein-Ligand Complex of Potential Leads

**Figure 4:**
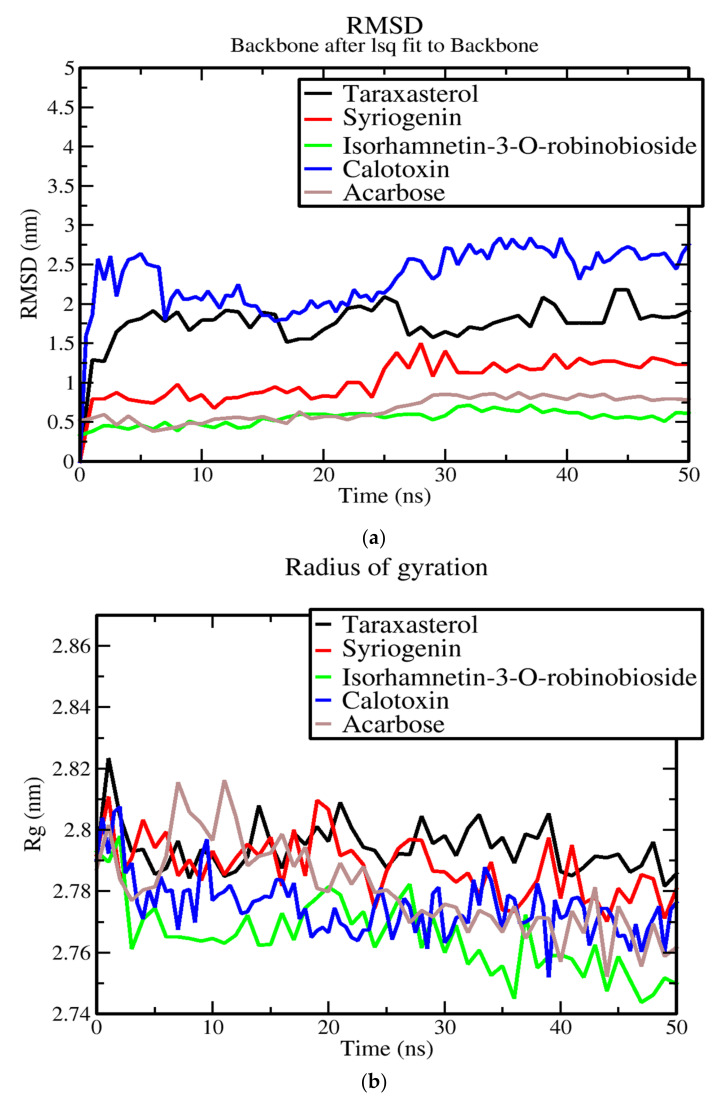

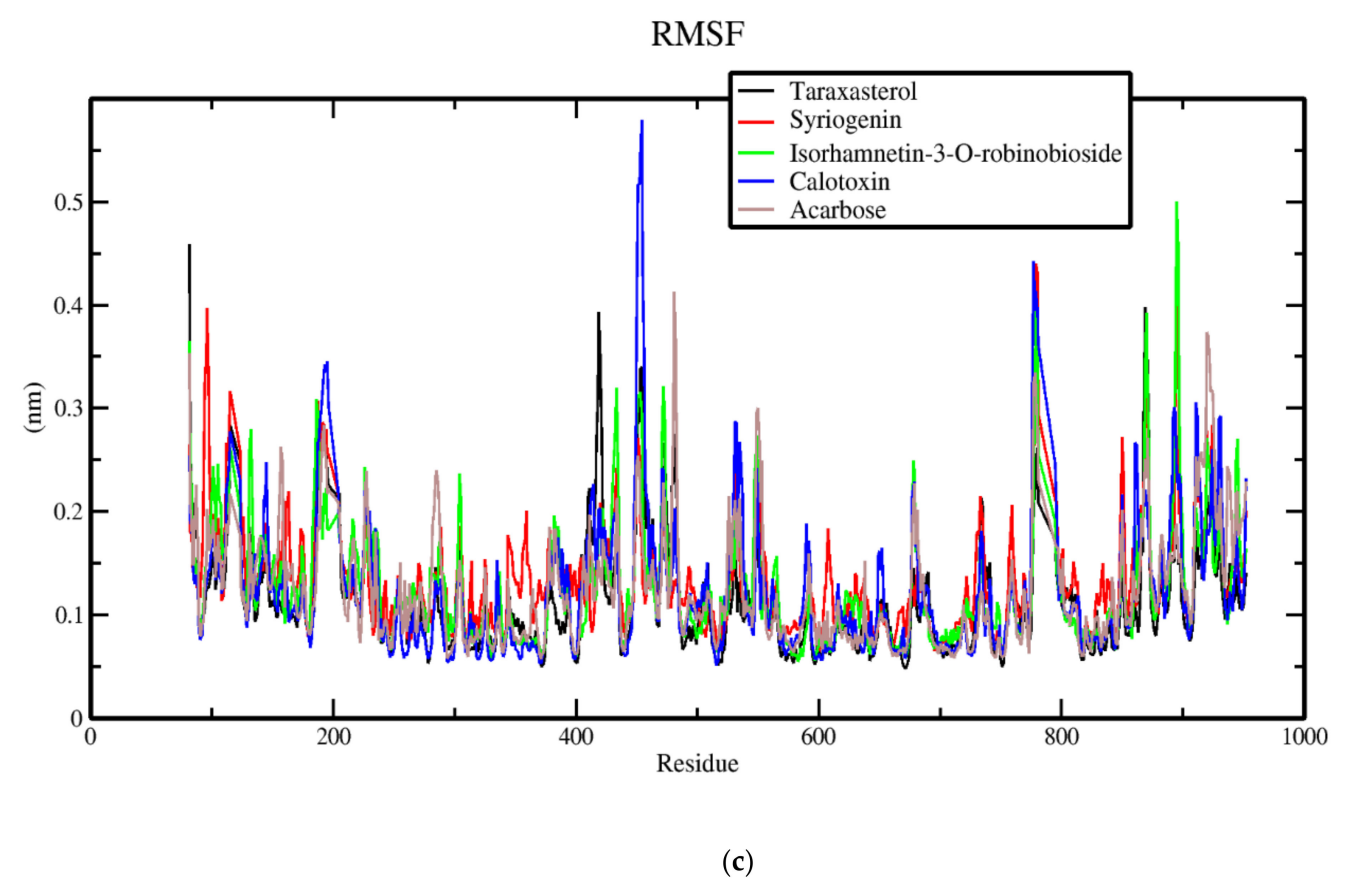
Graphs of RMSD, Rg and RMSF of alpha glucosidase-ligand complexes generated over 50 ns simulation using GROMACS: (**a**) radius of gyration versus time graph of the AG-ligand complexes, (**b**) RMSD versus time graph of the backbone atoms of AG-ligand complexes over 50 ns and (**c**) analysis of RMSF trajectories of residues of AG-ligand complexes. The four potential leads taraxasterol, syriogenin, isorhamnetin-3-O-robinobioside and calotoxin are color coded as black, red, green and blue, respectively. Acarbose used as a control is color coded as brown.

Molecular dynamics simulations are usually used to refine the results of docking methods for receptor-inhibitor complexes [6,97,98]. Molecular dynamics simulations are used to understand the dynamic features of the best-docked compounds with appropriate number of interactions with respect to time at a nanosecond scale [99]. Statistical parameters, such as RMSD, root mean square fluctuation (RMSF) and radius of gyrations (Rg), are used to further explain the results from the simulations [100]. RMSD values show how the backbone atoms of the enzymes and the ligands deviates and low RMSD values are an indication of stability of the complex [101]. RMSF is an important factor that provides information about structural flexibility of atoms in the system. The RMSF of a region is the average displacement of that region with respect to a reference position taken over the trajectory time [102]. The fluctuations of the protein-ligand complexes and ligand associated movements were analyzed within the hydrated system to check for movement and structural stability during the course of the simulation. This movement and stability are significant for the complex functioning inside living systems. Figure 4a depicts the RMSD of the protein-ligand complex simulations of taraxasterol, syriogenin, isorhamnetin-3-O-robinobioside, calotoxin and acarbose within 50 ns. The backbones of the complexes were stable after 25 ns and there was a general uniform stability from the period of 45 to 50 ns. Relatively, isorhamnetin-3-O-robinobioside complex had the lowest RMSD of 0.50 nm which stabilized between 30 and 50 ns. Acarbose complex had a closer RMSD of 0.75 nm with stabilization around 30–50 ns. Syriogenin had the third closest RMSD of 1.25 nm to acarbose followed by the AG complex of taraxasterol with an RMSD 1.80 nm, and lastly calotoxin with the highest RMSD 2.75. These also showed stabilization around 30–50 ns. The greater RMSD of calotoxin could contribute to instability in the protein complex. Apparently, inaccuracies in the model have a large impact in the quality of the simulation results. Instead, results clearly indicate that deviation from the original structure can be directly correlated with the loss of quality of the model [103]. The compactness of the complexes was determined by using the Rg. A stable folded protein is likely to maintain a relatively steady Rg [104]. In Figure 4b, the Rg values of all complexes gave an indication that the complexes remained stable over 50 ns. The Rg values of all complexes experienced a gradual fall throughout 10–50 ns. The Rg value of the acarbose complex was unstable during the first 20 ns. The instability propagated throughout to about 40 ns, until it became fairly stable in the final 10 ns. Taraxasterol had the highest Rg value around 2.78 nm whilst iorhamnetin-3-O-robinobioside experienced the lowest Rg compared to the other complexes. Syriogenin and calotoxin complexes had Rg values hovering around 2.78 nm over the course of the 50 ns simulation. To explore the flexibility of residues contribution to the structural fluctuation, RMSFs of each residue were assessed. The results of RMSFs showed consistency for the docked complexes (Figure 4c). Calotoxin complex exhibited the highest fluctuations around residue numbers 190–200, followed by acarbose and isorhamnetin-3-O-robinobioside. Further significant fluctuations occurred around residues 451–456, 776–781, 869–871 and 890–897. Overall, calotoxin complex showed the highest fluctuations around these residue regions followed by isorhamnetin-3-O-robinobioside. All the complexes exhibited some degree of fluctuations in these regions of the alpha glucosidase protein. A deduction from this trajectory profile assumes possible residues to include Tyr^191^, Glu^192^, Val^193^, Ala^452^, Gly^453^, Ser^454^, Tyr^455^, Arg^456^, Gln^776^, Tyr^777^, Va^778^, Pro^779^, Glu^869^, Arg^870^, Gly^871^, Ser^894^, Glu^895^, Gly^896^ and Ala^897^ to be responsible for such fluctuations. The region holding the highest RMSF values of 0.5 nm were 451–456. Followed by residues ranging from 776 to 781 above 0.4 nm. Higher RMSF values imply greater fluctuations. Greater amounts of structural fluctuation occur in regions known to be involved in ligand binding and catalysis, notably, the catalytic loop region [105]. Adaptive variations also lie in these regions which also contribute to stability of the complex [105].

### 3.8. Evaluation of Putative Leads Using MM-PBSA Approach

Typical scoring functions of molecular docking are limited partly by the treatment of solvation effects [106]. Physics-based scoring functions such as MM-PBSA are used to address this problem [107]. MM-PBSA was employed in determining the binding free energies of the complexes. Free energies drive all molecular processes such as protein folding, molecular association and chemical reactions [107]. Table 4 provides information on the contributing energies in the simulation. The compounds considered had diverse range of binding affinity towards alpha glucosidase in terms of binding free energies. Isorhamnetin-3-O-robinobioside had the lowest binding free energy of −111.99 ± 30.828 kJ/mol, followed by calotoxin (−83.963 ± 47.232 kJ/mol), syriogenin (−83.139 ± 16.039 kJ/mol) and taraxasterol (−80.125 ± 15.326 kJ/mol). Acarbose on the other hand, had a high binding free energy (513.34 ± 35.886 kJ/mol) and a good Van der Waals energy (−155.148 ± 26.589 kJ/mol). In addition to that, it had high polar solvation energy of 272.582 ± 49.072 kJ/mol and high electrostatic energy (413.658 ± 50.519 kJ/mol). This might possibly be due to the presence of many hydrophobic interactions. Van der Waals’s energy is usually a result of the temporary dipole formed between hydrophobic functional groups [108], thus it may be assumed that the hydrophobic interactions appear to be crucial in binding and stabilization of acarbose at the binding site. Compounds with high binding energies can be active against the target receptor due to high polar solvation energies [109]. From the molecular docking results, acarbose had binding energy of −34.3 kJ/mol. MM-PBSA results showed it to have a high positive binding free energy of 513.34 ± 35.886 kJ/mol, implying a low binding affinity among the four lead compounds. Most of its hydrophobic residues could be seen to contribute a higher energy decomposition per residue including residues Asp^282^, Trp^481^ and Asp^616^, which forms hydrogen bonding with the ligand (Supplementary Figure S5). Docking results of taraxasterol, syriogenin, isorhamnetin-3-O-robinobioside and calotoxin showed estimated binding energies of −40.2, −35.1, −34.3 and −34.3 kJ/mol, respectively. MM-PBSA calculations reinforced these predicted binding energies of the compounds against the alpha glucosidase receptor with correspondingly low free binding energies. Hence, improvement of the compound’s overall affinity. 

**Figure 5:**
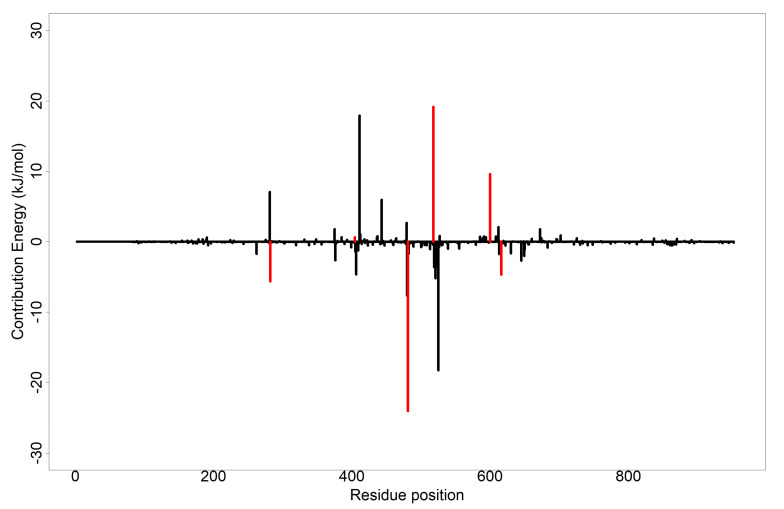
MM-PBSA plot showing the per-residue decomposition of the binding free energy of isorhamnetin-3-O-robinobioside in complex with alpha glucosidase. Red−colored tick extensions depict predicted hydrogen bonding residues.

To further understand the binding mechanisms, the total binding energy decomposition per-residue was analyzed [109,110]. Details of the results for the per-residue decomposition energy analysis for each inhibitor can be found in Supplementary Table S3 and Figures S2–S5. A residue whose total energy contribution is less than or equal to −4.5 kJ/mol and greater than or equal to 5 kJ/mol is considered crucial, thus corroborating the affinity of the ligand to the protein target [111,112]. Figure 5 shows the MM-PBSA analysis of the per-residue decomposition of the isorhamnetin-3-O-robinobioside complex with Asp^282^, Trp^481^, Asp^518^, Arg^600^ and Asp^616^ showing total energy contributions of −5.61, −24.04, 19.19, 9.63 and −4.67 kJ/mol, respectively. Asp^518^ and Arg^600^ exhibited total energy contribution values greater than or equal to −4.5 kJ/mol. Asp^282^, Trp^481^ and Asp^616^ contributed low total energy to isorhamnetin-3-O-robinobioside. From the LigPlot^+^ results (Table 2 and Table S2), Asp^282^ and Asp^616^ respectively contributed hydrogen bonds with lengths of 2.82 Å and 2.99 Å, except for Trp^481^ with bond length of 3.32 Å, which was above the reasonable length of 3.0 Å. Residues Trp^481^ and Phe^525^ contributed −13.10 kJ/mol and −9.5195 kJ/mol in the syriogenin-receptor complex, respectively. MM-PBSA plot of taraxasterol and syriogenin showed no residue contributing significantly to the binding free energy to be warranted as critical (Supplementary Figures S2 and S3). Complex of calotoxin with AG had residues such as leu^677^, Leu^678^ and leu^680^ with −4.5239, −5.9533 and −5.3047 kJ/mol contributing to the energy decomposition, respectively. None of the hydrogen bonding residues in calotoxin-AG complex contributed a significant energy decomposition in the complex. The MM-PBSA plot of the free binding energy contribution per-residue of the acarbose complex is shown in Supplementary Figure S5. Most of the hydrogen bond forming residues contributed higher residue decomposition energies. This included Asp^282^, Asp^404^ and Asp^616^ with corresponding energies of 17.0038, 19.5988 and 31.1002 kJ/mol, respectively. On the other hand, less energy residue decomposition of −8.3071 was contributed by Arg^600^. In all, the complex of calotoxin showed possible critical residues as leu^677^ (−4.5239 kJ/mol), Leu^678^ (−5.9533 kJ/mol), leu680 (−5.3047 kJ/mol); and isorhamnetin-3-O-robinobioside with Trp^481^ (−24.04 kJ/mol) and Asp^518^ (19.19 kJ/mol). It appears there is no published evidence regarding the importance of these residues in the binding mechanisms of alpha glucosidase. However, that of Asp^282^, Arg^600^ and Asp^616^ observed in isorhamnetin-3-O-robinobioside have been addressed as critical to binding of alpha glucosidase [37]. Apart from identifying crucial amino acids, the results of the per-residue decomposition energy analysis clarified the individual energy contributions of all the amino acid residues.

### 3.9. Exploring Possible Structural Similarity of Predicted Leads

An exhaustive search of structurally similar compounds to the potential leads, purported to show antidiabetic activity was done via the DrugBank database [113]. The SMILES file of each compound was used as query for structural similarity using a Tanimoto coefficient of 0.70. Tanimoto coefficient score between 0.70 and 1.0 is an indication of a higher similarity between two compounds [114]. Table 5 provides the IUPAC names used for the similarity search together with their 2D structures. Isorhamnetic-3-O-robinobioside had similarity scores of 0.987 and 0.979 for rutin and isoquercetin, respectively. These compounds belong to the class of organic compounds known as flavonoid-3-o-glycosides. These are phenolic compounds containing a flavonoid moiety which is O-glycosidically linked to carbohydrate moiety at the C3-position. Isoquercetin amongst other two flavonoids were evaluated as alpha-glucosidase inhibitors by fluorescence spectroscopy and enzymatic kinetics and were also been compared with acarbose [115]. Rutin on the other hand is an approved drug that has been used therapeutically to decrease capillary fragility [116], whilst isoquercetin remains under investigation. Taraxasterol had a similarity of 0.889 with lupeol, an investigational drug belonging to the class of organic compounds known as triterpenoids. This has been shown together with another two triterpenoids to inhibit the α-glucosidase enzyme in a concentration-dependent manner, and their inhibitory activity was higher than that of the antidiabetic drug acarbose (IC_50_ 241.6 µM) [117]. Kinetic analysis established that lupeol acted as competitive inhibitor and further docking analysis suggested that all three triterpenes (betulinic acid, botulin and lupeol) bind at the same site as acarbose does in the human intestinal α-glucosidase [117]. Moreover, lupeol analogues containing a benzylidene chain exhibited the best activity against *α*-glucosidase and better IC_50_ values to the positive agent (acarbose) [118].

The remaining potential lead compounds syriogenin and calotoxin showed similarities with other compounds, such as digoxigenin and peruvoside, which had no related effect against alpha glucosidase or antidiabetic activity. They however fall under the class of steroids. Some steroids nonetheless, such as ergosterols and beta-sitosterol, possess good activity against alpha glucosidase [70]. As a result, these compounds could be worthwhile considering as potential lead compounds against alpha glucosidase.

## 4. Conclusions

Alpha glucosidase inhibition with natural products is of utmost significance when it comes to mitigating DM. Inhibition of this target leads to the control of hyperglycemia in DM and its complications. Taraxasterol, syriogenin, isorhamnetin-3-O-robinobioside and calotoxin, are compounds reportedly isolated from the *Calotropis procera* plant. These compounds were identified as potential inhibitors of alpha glucosidase through cheminformatics studies. These compounds have appreciably high binding affinity to the receptor and were also predicted to possess alpha-glucosidase activity. Residues such as Trp^481^, Asp^518^, Leu^677^, Leu^678^ and Leu^680^, were observed to contribute substantial energies critical for binding via the MM-PBSA per-residue decomposition analysis, hence making them crucial for the binding mechanisms of alpha glucosidase. Isorhamnetin-3-O-robinobioside was assessed to be similar to that of acarbose with respect to its high binding affinity, hydrogen bonds interactions, bond lengths, lowest free binding energy and five interacting residues elucidated via energy decomposition. Notwithstanding, these four compounds are potential novel leads which require both in vitro and in vivo evaluations of their effect on alpha glucosidase activity. Although, the study was entirely computational, these compounds can be explored as the basis for designing of potent inhibitors of alpha glucosidase.

## Figures and Tables

**Table 1:** Reported isolated compounds from different parts of *Calotropis procera*.

No.	Compound Names	Sources	PubChem ID	Mol. Weight	Refs.
1	Isorhamnetin-3-O-rutinoside	Stem/Latex	5481663	624.54	[19]
2	Isorhamnetin-3-O-robinobioside	Stem/Latex	5491808	624.5	[19,20]
3	Calotropagenin	Leaf/Latex	212348	404.5	[18]
4	Calotoxin	Latex	56840852	404.5	[19,22]
5	Uscharin	Latex/Leaf	11261800	587.72	[19,25]
6	Voruscharin	Latex	44387915	589.74	[19]
7	2,7,10-trimethyldodecane	Stem Bark	93447	212.41	[19]
8	Luteolin	Leaf	15661823	300.26	[19]
9	Ursolic Acid	Leaf	64945	456.7	[19]
10	β-amyrin	Latex/Root	73145	426.72	[19,45]
11	Syriogenin	Leaf	11870470	390.51	[18,19]
12	Lactucerol	Latex	115250	426.7	[19]
13	Octadecenamide	Stem Bark	6443016	281.5	[19]
14	Z-13 docosinamide	Stem/Latex	5365371	337.6	[19]
15	Tyranton	Leaf	31256	116.16	[19]
16	1-heptadecene	Leaf	23217	238.5	[19]
17	Taraxasterol	Root	344468	468.8	[19,45]
18	Benzoyllineolone	Root bark	5322013	468.6	[19,45]
19	3-epimoretenol	Latex	604951	426.72	[19]
20	1-pentadecene	Leaf	25913	210.4	[19]
21	Isobutylnonane	Stem/Latex	545936	184.36	[19]
22	α-amyrin	Root bark	73170	426.72	[19,45]
23	Glibenclamide	Root	3488	494	[24]
24	Apigenin-7-0-glucoside	Leaf/Root	5280704	432.38	[18,19]
25	Thioacetic acid	Leaf	10484	76.12	[18,19]
26	kaempferol-7-0-glucoside	Leaf	10095180	448.38	[19,25]
27	Quercetin-3-rutinoside	Latex	5280805	610.5	[19]
28	Calotropin	Leaf/Stem/latex	16142	532.6	[19]
29	Beta sitosterol	Stem Bark	222284	414.71	[19,25]
30	Benzoylisolineolone	Root bark	9982084	468.58	[19]
31	Calactin	Leaf	-	523.6	[19]
32	Procesterol	Undried flower	-	428.69	[46]

**Table 2:** Binding energies of extracted compounds and acarbose with their corresponding hydrogen and hydrophobic interacting residues.

Extracted Compounds	Binding Energies (kJ/mol)	Hydrogen Bonding Interacting Residues and Bond Lengths (Å)	Hydrophobic Bond Interacting Residues
Taraxasterol	−40.2	Leu^677^ (3.07), Leu^678^ (3.32)	Asp^282^, Leu^283^, Ala^284^, Trp^376^, Trp^481^, Met^519^, Ser^523^, Phe^525^, Asp^616^, Phe^649^, Leu^650^, Ser^676^
Voruscharin	−39.3	Arg^281^ (3.07), Asp^616^ (2.36), Leu^678^ (3.23)	Asp^282^, Trp^376^, Trp^481^, Met^519^, Ala^655^, Phe^649^, Leu^650^, Ser^676^, Leu^677^
Alpha-amyrin	−37.7	Phe^525^ (3.17)	Asp^282^, Trp^376^, Trp^481^, Ser^523^, Asp^524^, Ala^555^, Asp^616^, Leu^650^, Phe^649^
3-epimoretenol	−36.8	None	Asp^282^, Trp^376^, Trp^481^, Met^519^, Asn^524^, Phe^525^, Phe^649^
Lactucerol	−36.4	None	Asp^282^, Trp^376^, Trp4^81^, Asn^524^, Phe525, Ala^555^, Phe^649^, Leu^650^, Ser^676^
Beta-sitosterol	−36.4	Asn^524^ (3.11)	Asp^282^, Trp^376^, Leu^404^, Trp^481^, Ser^523^, Asn^524^, Phe^525^, Ala^555^, Asp^616^, Phe^649^, Leu^650^, Ser^676^
Beta-amyrin	−36.0	None	Asp^282^, Trp^376^, Trp^481^, Asn^524^, Phe^525^, Ala^555^, Phe^649^, Leu^650^, Asp^616^, Ser^676^
Apigenin-7-0-glucoside	−36.0	Asp^404^ (2.61, 2.94), Asn^524^ (2.92), Arg^600^ (2.94, 3.17), Asp^616^ (2.87, 3.30), His^674^ (3.22)	Asp^282^, Trp^376^, Leu^405^, Trp^481^, Ile^441^, Asp^518^, Met^519^, Phe^525^, Ala^555^, Phe^649^
Uscharin	−35.1	Asp^616^ (2.76)	Asp^282^, Trp^376^, Trp^481^, Asn^524^, Phe^525^, Phe^649^, Leu^650^, Asp^616^, Ser^676^, Leu^677^, Leu^678^
Syriogenin	−35.1	Arg^281^ (3.16), Asp^282^ (3.10), Asp^616^ (2.88), Leu^677^ (3.27)	Trp^376^, Trp^481^, Met^519^, Asn^524^, Phe^525^, Ala^555^, Phe^649^, Leu^650^, Phe^649^, Leu^650^, Ser^676^
Quercetin-3-rutinoside	−34.7	Asp^282^ (2.74,3.15,3.16), Asp^404^ (2.44), Asp^518^ (2.94), Ser^523^ (3.08), Arg^600^ (2.67, 3.15), Asp^616^ (3.04, 3.19), His^674^ (2.91)	Leu^283^, Ala^284^, Trp^376^, Trp^481^, Trp^516^, Met^519^, Asn^524^, Phe^525^, Phe^649^, Leu^650^
Glibenclamide	−34.7	Arg^281^ (3.02), Asp^616^ (2.85, 3.01)	Asp^282^, Leu^283^, Trp^376^, Asp^404^, Ile^441^, Trp^481^, Asn^524^, Phe^525^, Asp^518^, Ala^555^, Phe^649^, His^674^
Benzoyllineolone	−34.7	Asp^282^ (2.81)	Leu^283^, Ala^284^, Trp^376^, Trp^481^, Phe^525^, Ala^555^, Asp^616^, Phe^649^, Leu^650^
Kaempferol-7-0-glucoside	−34.3	Arg^281^ (3.20), Asp^282^ (2.91), Asp^404^ (3.02), Ser^523^ (3.07, 2.74), Asn^524^ (2.70, 3.00)	Leu^283^, Trp^376^, Ile^441^, Trp^481^, Phe^525^, Asp^518^, Trp^516^, Met^519^, Ala^555^, Asp^616^, Phe^649^
Ursolic acid	−34.3	None	Asp^282^, Trp^376^, Trp^481^, Asn^518^, Phe^525^, Ala^555^, Arg^600^, Asp^616^, Phe^649^, Ser^676^
Isorhamnetin-3-O-rutinoside	−34.3	Asp^282^ (2.82), Asp^404^ (3.03), Trp^481^ (3.32), Asp^518^ (2.81, 3.07), Arg^600^ (3.25), Asp^616^ (2.99)	Asp^282^, Leu^283^, Trp^376^, Ile^441^, Trp^481^, Asn^524^, Phe^525^, Asp^518^, Ala^555^, Phe^649^, His^674^
Isorhamnetin-3-O-robinobioside	−34.3	Asp^282^ (2.82), Asp^404^ (3.03), Trp^481^ (3.32), Asp^518^ (2.81, 3.07), Arg^600^ (3.25), Asp^616^ (2.99)	Leu^283^, Trp^376^, Leu^405^, Ile^441^, Phe^525^, Trp^613^, Leu^650^, Se^r676^
Calotoxin	−34.3	Asp^282^ (3.15), Asn^524^ (2.86), Phe^525^ (2.79), Asp^616^ (2.71)	Arg^281^, Leu^283^, Ala^284^, Trp^376^, Ala^555^, Leu^650^
Acarbose	−34.3	Asp^282^ (2.78,2.82,2.99), Asp^404^ (2.70, 2.86), Asn^524^ (2.80), Phe^525^ (2.92), Arg^600^ (2.81, 2.83), Asp^616^ (2.70, 2.80), His^674^ (3.05)	Asp^281^, Leu^283^, Ala^284^, Trp^376^, Leu^405^, Ile^441^, Trp^481^, Trp^516^, Asp^518^, Met^519^, Ala^555^, Trp^613^, Phe^649^
Calactin	−33.5	Trp^618^ (3.17)	Arg^281^, Asp^282^, Ala^284^, Asn^524^, Phe^525^, Arg^527^, Ala^555^, Asp^616^, Leu^650^
Calotropin	−33.5	Trp^618^ (3.21)	Arg^281^, Asp^282^, Ala^284^, Asn^524^, Phe^525^, Arg^527^, Ala^555^, Asp^616^, Leu^650^
Procesterol	−33.1	Asp^282^ (2.91,3.11), Arg^600^ (2.99), Asp^616^ (3.14)	Trp^376^, Met^519^, Phe^525^, Trp^618^, Phe^649^, Leu^650^, Gly^651^, Ser^676^, Leu^677^, Leu^678^
Benzoylisolineolone	−33.1	Arg^281^ (3.16), Ala^284^ (2.99)	Asp^282^, Leu^283^, Ala^284^, Trp^376^, Phe^525^, Phe^649^, Leu^650^
Calotropagenin	−32.6	Asp^91^ (3.14), Asp^95^ (3.26)	Ala^93^, Lys^96^, Ala^97^, Ile^98^, Tyr^110^, Pro^125^, Trp^126^, Arg^275^
luteolin	−31.4	Asp^282^ (3.15), Asp^404^ (2.86), Ser^523^ (3.13), His^674^ (2.96)	Trp^376^, Trp^481^, Trp^516^, Asp^518^, Met^519^, Phe^525^, Asp^616^, Phe^649^
2,7,10-trimethyldodecane	−23.4	None	Trp^376^, Leu^405^, Trp^481^, Ile^441^, Asp^518^, Met^519^, Phe^525^, Ala^555^, Asp^616^, Phe^649^, Leu^677^
Octadecenamide	−21.3	Asp^518^ (3.23), Asp^616^ (3.26) His^674^ (3.16)	Trp^376^, Phe^525^, Trp^613^, Phe^649^, Leu^650^, Ser^676^ Leu^677^, Leu^678^
1-_pentadecene	−21.3	None	Trp^376^, Leu^405^, Trp^481^, Ile^441^, Asp^518^, Met^519^, Phe^525^, Ala^555^, Asp^616^, Phe^649^, Leu^677^
Z-13_docosinamide	−20.9	None	Asp^282^, Trp^376^, Leu^405^, Trp^481^, Ile^441^, Asp^518^, Met^519^, Phe^525^, Asp^616^, Phe^649^, Leu^677^
Isobutylnonane	−20.5	None	Asp^282^, Trp^376^, Asp^404^, Trp^481^, Asp^518^, Met^519^, Phe^525^, Arg^600^, Asp^616^, Phe^649^, Leu^677^
1-heptadecene	−19.7	None	Trp^376^, Asp^404^, Trp^481^, Asp^518^, Met^519^, Phe^525^, Arg^600^, Asp^616^, Phe^649^, Leu^677^
Tyranton	−19.2	Trp^481^ (3.21), Asp^518^ (2.92) Arg^600^ (3.03)	Trp^376^, Asp^404^, Leu^405^, Trp^481^, Trp^516^, Met^519^, Asp^616^, Phe^649^, His^674^
Thioacetic acid	−10.9	His^674^ (3.01)	Trp^516^, Asp^518^, Trp^613^, Asp^616^, Phe^649^

**Table 3:** Biological activity prediction results of nine selected compounds. Pa and Pi denotes probability of activity and inhibition, respectively. When Pa > Pi, the compound is attractive to be explored experimentally for the predicted activity.

Compound	Pa	Pi	Activity
Taraxasterol	0.200	0.005	α-Glucosidase inhibitor
	0.141	0.069	Antidiabetic type 1
	0.367	0.008	Hydroxysteroid dehydrogenase inhibitor
	0.332	0.009	Protein tyrosine phosphate inhibitor
	0.226	0.005	17-Beta-hydroxysterol dehydrogenase inhibitor
3-epimoretenol	0.142	0.012	Alpha glucosidase activity
	0.057	0.029	17-Beta-hydroxysterol dehydrogenase inhibitor
	0.128	0.113	Antidiabetic type 2
Lactucerol	0.200	0.050	α-Glucosidase inhibitor
Syriogenin	0.102	0.029	α-Glucosidase inhibitor
Isorhamnetin-3-O-robinobioside	0.818	0.001	α-Glucosidase inhibitor
Calotoxin	0.101	0.029	α-Glucosidase inhibitor

**Table 4:** The energy terms obtained after MM-PBSA analysis of the protein-ligand complexes. The energy values are presented as mean ± standard deviation (kJ/mol).

Compound	Van der Waals Energy	Electrostatic Energy	Polar Solvation Energy	SASA Energy	Binding Energy
Taraxasterol	−102.625 ± 17.227	−2.795 ± 6.568	35.103 ± 11.322	−9.808 ± 1.483	−80.125 ± 15.326
Syriogenin	−102.534 ± 13.538	−27.083 ± 21.100	56.247 ± 29.946	−9.769 ± 1.843	−83.139 ± 16.039
Isorhamnetin-3-O-robinobioside	−203.397 ± 18.850	−141.376 ± 24.067	252.953 ± 36.473	−20.17 ± 1.577	−111.99 ± 30.828
Calotoxin	−114.182 ± 24.776	−14.063 ± 18.510	55.190 ± 46.644	−10.91 ± 2.923	−83.963 ± 47.232
Acarbose	−155.148 ± 26.589	413.658 ± 50.519	272.582 ± 49.072	−17.75 ± 1.949	513.34 ± 35.886

**Table 5:** List of predicted lead compounds with their IUPAC names and 2D structures.

**Name of Compound**	**IUPAC Name**	**2D Structure**
Taraxasterol	(3S,4aR,6aR,6aR,6bR,8aR,12S,12aR,14aR,14bR)-4,4,6a,6b,8a,12,14b-heptamethyl-11-methylidene-1,2,3,4a,5,6,6a,7,8,9,10,12,12a,13,14,14a-hexadecahydropicen-3-ol	** 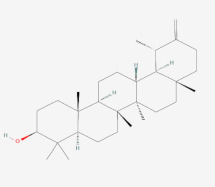 **
Syriogenin	3-[(3S,5S,8R,9S,10S,12R,13S,14S,17R)-3,12,14-trihydroxy-10,13-dimethyl-1,2,3,4,5,6,7,8,9,11,12,15,16,17-tetradecahydrocyclopenta[a]phenanthren-17-yl]-2H-furan-5-one	** 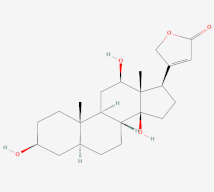 **
Isorhamnetin-3-O-robinobioside	5,7-dihydroxy-2-(4-hydroxy-3-methoxyphenyl)-3-[(3R,4S,5R,6R)-3,4,5-trihydroxy-6-[[(2R,3R,4R,5R,6S)-3,4,5-trihydroxy-6-methyloxan-2-yl]oxymethyl]oxan-2-yl]oxychromen-4-one	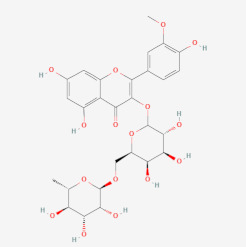
Calotoxin	(1S,3R,5S,7R,8S,9R,10S,12R,14R,18R,19R,22S,23R)-8,9,10,22-tetrahydroxy-7,18-dimethyl-19-(5-oxo-2H-furan-3-yl)-4,6,11-trioxahexacyclo [12.11.0.03,12.05,10.015,23.018,22]pentacosane-14-carbaldehyde	** 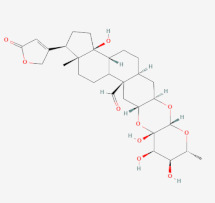 **

## Data Availability

Not applicable.

## Supplementary Materials

The following supporting information can be downloaded at: https://www.mdpi.com/article/10.3390/cimb44020064/s1, Figure S1. RMSD of 5NN8during molecular dynamics simulations for 50 ns, Figure S2. MM-PBSA plot of free binding energy contribution per residue decomposition of taraxasterol-alpha glucosidase complex, Figure S3. MM-PBSA plot of free binding energy contribution per residue decomposition of syriogenin-alpha glucosidase complex. Hydrogen bonding residues labelled in red, Figure S4. MM-PBSA plot of free binding energy contribution per residue of the complex of calotoxin with alpha glucosidase. Hydrogen bonding residues labelled in red, Figure S5. MM-PBSA plot of free binding energy contribution per residue of acarbose-alpha glucosidase complex. Hydrogen bonding residues labelled in red, Table S1. Some selected known inhibitors of alpha glucosidase obtained from literature used in screening against alpha glucosidase and their corresponding references [119,120], Table S2. Binding affinities and molecular interactions between selected known inhibitors and alpha glucosidase (5nn8), Table S3. Per-residue decomposition analysis of amino acids of the potential lead compounds.
