# Supplementary material for: Molecular Structure-Based Screening of the Constituents of Calotropis procera Identifies Potential Inhibitors of Diabetes Mellitus Target Alpha Glucosidase
Source: Curr Issues Mol Biol. 2022 Feb 21;44(2):963–87. doi: 10.3390/cimb44020064 (PMC8928985; doi:10.3390/cimb44020064)
Supplement: Supplementary file 1 [file cimb-44-00064-s001.zip › cimb-1549308-supplementary.pdf]

Supplementary Information

Table S1: Some selected known Inhibitors of alpha glucosidase obtained from literature used in screening against alpha glucosidase and their corresponding references.

Compound	IC_{50}	Unit	Reference
Acacetin	0.16	mmol/L	[1]
Kaempferol	1.16 ± 0.04 x10^5	mol/L	[2]
	153.39 ± 1.79	μM	[3]
Hesperetin 5-O-glucoside	41.25 ± 1.25	μg/mL	[4]
Luteolin	82.73 ± 2.51	μM	[3]
Rutin	0.196	mM	[5]
	173.58 ± 1.23	μM	[3]
Plicatanoside	111.23 ± 0.65	uM	[6]
Sulochrin	8.5	mg/mL	[7]
Vitexin	0.42	mmol/L	[1]
Isoquercetrin	0.185	mM	[5]
	0.24	mmol/L	[1]
Rosmarinic acid	33.0 ± 4.6	μmol/L	[1]
Swertisin	0.37	mmol/L	[1]
Acteoside	1.62 ± 0.29	mmol/L	[1]
Acarbose	2.09 ± 0.03 x 10^4	mol/L	[1]
	0.59 ± 0.14	μM	[1]
	996	nM	[8]
Fisetin	19,600	nM	[8]
Quercetin	0.017	mM	[5]
	14.6 ± 3.72	μmol/L	[1]
Myricetin	11.63 ± 0.36	μM	[3]
	30,200	nM	[8]
4-(p-toluenesulfonamide)-3,4-dihydroxy chalcone	193,700	nM	[8]
4-Amino-4-hydroxychalcone	268,900	nM	[8]
Apigenen-7-O-rutinoside	64.29 ± 2.41	μg/mL	[9]
Miglitol	465.1	μM	[10]
Naringenin	44.65	μg/mL	[11]
Tiliroside	14.5 ± 0.1	μmol/L	[1]
verbascoside	0.5 ± 0.03	mmol/L	[1]
Leucosceptoside	0.7 ± 0.04	mmol/L	[1]
Isoacteoside	0.1 ± 0.01	mmol/L	[1]

Table S2. Binding affinities and molecular interactions between selected known inhibitors and alpha glucosidase (5nn8).

**Known Inibitors compounds**	**Binding Energy kJ/mol**	**Hydrogen bonding residues and bond length Å**	**Hydrophobic bonding residues**
Apigenin-7-O-Rutinoside	-38.1	Tyr^{360}(3.08)<br>Met^{363}(3.04)<br>His^{584}(3.11)<br>Arg^{594}(3.07)<br>Arg^{608}(2.84, 3.09)<br>Ser^{864}(2.93)	Gly^{359}, Phe^{362}, Tyr^{609}, His^{717}, Leu^{865}, Glu^{866} Val^{867}, Leu^{868}, Glu^{869}
Plicatanoside	-37.7	Asp^{356}(2.85)<br>Arg^{608}(2.70, 3.00)<br>Glu^{869}(3.22)	Leu^{355}, Gly^{359}, Phe^{362}, His^{584}, Val^{718} His^{717}, Leu^{860}, Leu^{865}, Glu^{866}, Val^{867}, Leu^{868}, Glu^{869}
Rutin	-37.7	Trp^{360}(2.67)<br>Leu^{355}(2.91)<br>Asp^{356}(2.85)<br>Met^{363}(3.22)<br>Arg^{608}(2.89,3.10,3.10)	Pro^{361}, Phe^{362}, Gly^{359}, Val^{718} His^{717}, Leu^{860}, Leu^{865}, Glu^{866} Val^{867}, Leu^{868}
Leucosceptoside	-36.0	Asp^{365}(2.99)<br>His^{584}(3.12)<br>Arg^{608}(2.81)<br>His^{717}(2.92)<br>Ser^{864}(2.92)<br>Val^{867}(2.99)	Try^{360}, Gly^{359}, Met^{363}, Pro^{595}, Glu^{866}, Val^{867}, Leu^{868}, Glu^{869}
Vitexin	-35.6	Tyr^{360}(2.70)<br>Met^{363}(3.12)<br>Arg^{608}(3.26, 3.30)<br>Val^{867}(2.80)<br>Leu^{868}(3.00)	Pro^{361}, Phe^{362}, His^{584}, Arg^{594}, Tyr^{609}, His^{717}, Leu^{865}, Glu^{866}, Glu^{869}
Isoacteoside	-35.1	Pro^{94}(2.93, 3.27)<br>Asp^{95}(2.98)<br>Arg^{275}(3.15)<br>Arg^{331}(2.79, 2.92)<br>Tyr^{543}(3.17)<br>Val^{544}(2.92, 3.15)	Asp^{91}, Ile^{98}, Tyr^{110}, Gly^{123}, Gln^{124}, Pro1^{25}, Cys^{127}, Val^{321}
Verbascoside	-35.1	Asp^{356}(2.95,3.17)<br>Arg^{608}(2.76)<br>His^{717}(3.03)<br>Ser^{864}(2.80)<br>Va^{l867}(3.03)	Try^{360}, Gly^{359}, Met^{363}, His^{584}, Arg^{594}, Pro^{595}, Glu^{866}, Val^{867}, Leu^{868}, Glu^{869}
Hesperetin_5-O-glucoside	-34.3	Ile581(2.86)<br>Arg^{608}(3.25)<br>Ser^{864}(2.79)<br>Leu^{868}(3.04,3.26)<br>Glu^{869}(3.20)	Pro^{361}, Met^{363}, Phe^{362}, His^{584}, Arg^{585}, Arg^{594}, Tyr^{609}, His^{717}, Leu^{865},
Swertisin	-33.9	Arg^{594}(3.01)<br>Ser^{864}(2.84)<br>Glu^{866}(2.97)<br>Leu^{868}(3.05, 3.07)<br>Glu^{869}(2.97)	Phe^{362}, His^{584}, Arg^{585}, His^{717}, Leu^{865}, Val^{867}

Tiliroside	-33.5	Tyr^{360}(2.67)<br>His^{584}(2.89)<br>His^{717}(2.99)<br>Arg^{608}(3.10)<br>Glu^{866}(2.72)<br>Leu^{868}(2.86)<br>Glu^{869}(3.08)	Pro^{361}, Phe^{362}, Arg^{594}, Leu^{865}, Val^{867}
Isoquercetrin	-33.1	Asp^{91}(3.17)<br>Glu^{124}(2.91)<br>Trp^{273}(2.70, 3.00)<br>Arg^{331}(2.84, 3.60)<br>Tyr^{543}(3.20, 3.21)	Ala^{93}, Ile^{98}, Tyr^{110}, Gly^{123}, Gln^{124}, Pro^{125}, Trp^{126}, Cys^{127}, Trp^{274}, Val^{321},
Fisetin	-31.4	Tyr^{360}(2.17, 3.21)<br>His^{584}(2.94)<br>Val^{867}(3.08)	Phe362, Arg594, Arg608, His717, Ser864, Leu865, Glu866
Luteolin	-31.4	Asp^{282}(3.13)<br>Asp^{404}(3.03)<br>Ser^{523}(3.09)<br>His^{674}(2.92)	Trp^{376}, Trp^{481}, Trp^{516}, Asp^{518}, Met^{519}, Phe^{525}, Asp^{616}, Phe^{649}
Myricetin	-31.4	Tyr^{360}(2.90, 3.07)<br>His^{584}(2.89)<br>Arg^{608}(2.85)<br>Val^{867}(3.16)	Phe^{362}, Arg^{594}, Pro^{595}, His^{717}, Leu^{865}, Glu^{866}
Quercetin	-31.4	Tyr^{360}(2.88)<br>His^{584}(3.06)<br>Arg^{608}(2.91)<br>Val^{867}(2.88,3.15)	Phe^{362}, Arg^{594}, Pro^{595}, His^{717}, Leu^{865}, Glu^{866}
Sulochrin	-31.0	Met^{363}(2.86)<br>His^{584}(3.30)<br>Val^{867}(3.00)	Phe^{362}, Arg^{594}, Arg^{608}<br>His^{717}, Asp^{860}, Leu^{865}, Glu^{866}, Val^{867},Leu^{868}
Acacetin	-30.1	Leu^{756}(2.91)	Val^{740}, His^{742}, Thr^{739}, Gln^{743}, Val^{755}, Gln^{757}, Lys^{760}, Glu^{762}, Thr^{764}, Trp^{804}
Naringenin	-30.1	Leu^{756}(2.91)	Val^{740}, His^{742}, Thr^{739}, Gln^{743}, Val^{755}, Gln^{757}, Lys^{760}, Glu^{762}, Thr^{764}, Trp^{804}
Rosmaric_acid	-30.1	His^{584}(2.95)<br>Arg^{608}(2.95,3.26)<br>Val^{867}(2.85)<br>Leu^{868}(2.95)<br>Gln^{869}(3.24)	Phe^{362}, Met^{363}, Arg^{594}, Pro^{595}, Tyr^{609}, His^{717}, Ser^{864}, Leu^{865}, Glu^{866}
Kaempferol	-29.3	Leu^{756}(3.01)	Thr^{739}, Gln^{743}, Val^{755}, Gln^{757}, Lys^{760}, Glu^{762}, Thr^{764}, Trp^{804}
4'-Amino-4-hydroxychalcone	-28.03	Asp^{518}(2.87)	Trp^{376}, Asp^{404}, Trp^{516}, Arg^{600}, Asp^{616}, Phe^{649}, Leu^{677}
Voglibose	-24.7	Met^{363}(2.78)<br>His^{584}(3.02)<br>Ser^{864}(3.04)<br>Val^{867}(3.16)<br>Leu^{868}(2.87)	Phe^{362}, Arg^{594}, Pro^{595}, His^{717}, Leu^{865}, Glu^{866},
Miglitol	-23.4	Met^{363}(2.89, 2.96)<br>His^{717}(3.01)<br>Ser^{864}(2.70)	Phe^{362}, Arg^{594}, Pro^{595}, Leu^{865}, Glu^{866}

		Val^{867}(3.17)<br>Leu^{868}(3.17,3.03)	
4-(p-toluensesul fonamide)-3,4-dihydroxy chalcone	-23.4	Val^{236}(3.00)	Val^{84}, Phe^{90}, Phe^{129}, Pro1^{30}, Pro^{131}, Pro^{217}, ^{Ala237}, Pro^{238}

Table S3. Per – residue decomposition analysis of amino acids of the potential lead compounds.

Compound	H-bond Residue	Total Energy (kJ/mol)
Taraxasterol	Leu^{677}	-0.0050
	Leu^{687}	-0.0075
Syriogenin	Asp^{281}	0.3686
	Asp^{282}	-0.4018
	Asp^{616}	-0.3891
	Leu^{677}	-0.0750
Isorhamnetin-3-O-robinobioside	Asp^{282}	-5.61
	Asp^{404}	0.63
	Trp^{481}	-24.04
	Asp^{518}	19.19
	Arg^{600}	9.63
	Asp^{616}	-4.67
Calotoxin	Asp^{282},	0.1122
	Asn^{524}	0.0073
	Phe^{525}	-0.0382
	Asp^{616}	-0.0404
Acarbose	Asp^{282}	17.0038
	Asp^{404}	19.5988
	Asn^{524}	0.2110
	Phe^{525}	-0.3763
	Arg^{600}	-8.3071
	Asp^{616}	31.1002
	His^{674}	-2.2183

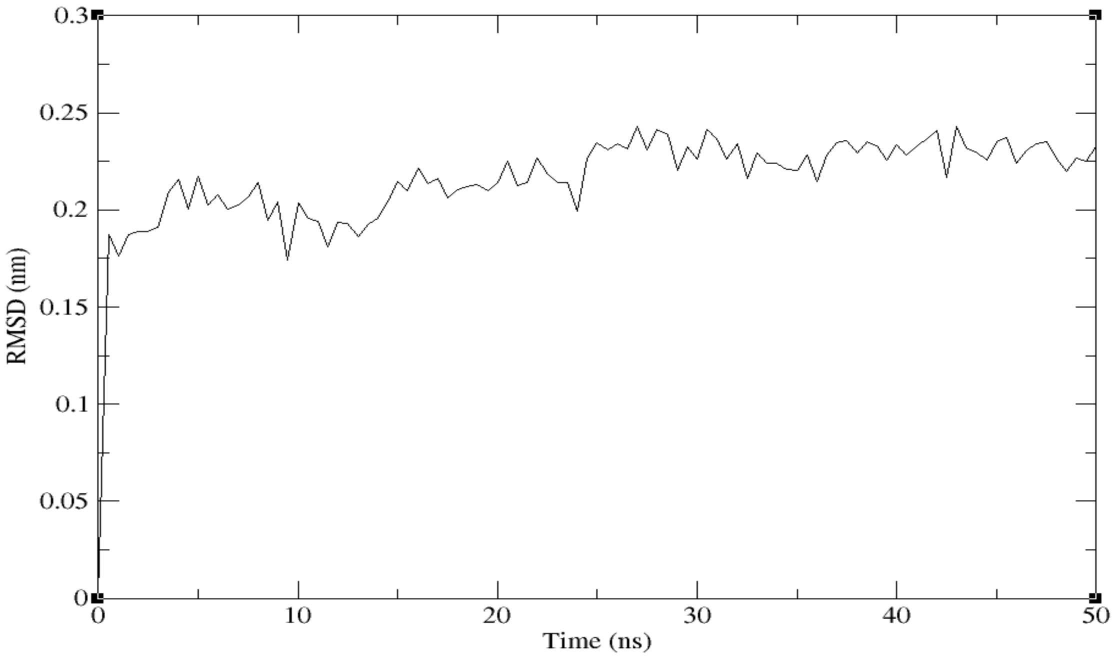

Figure S1. RMSD of 5nn8 during molecular dynamics simulations for 50ns.

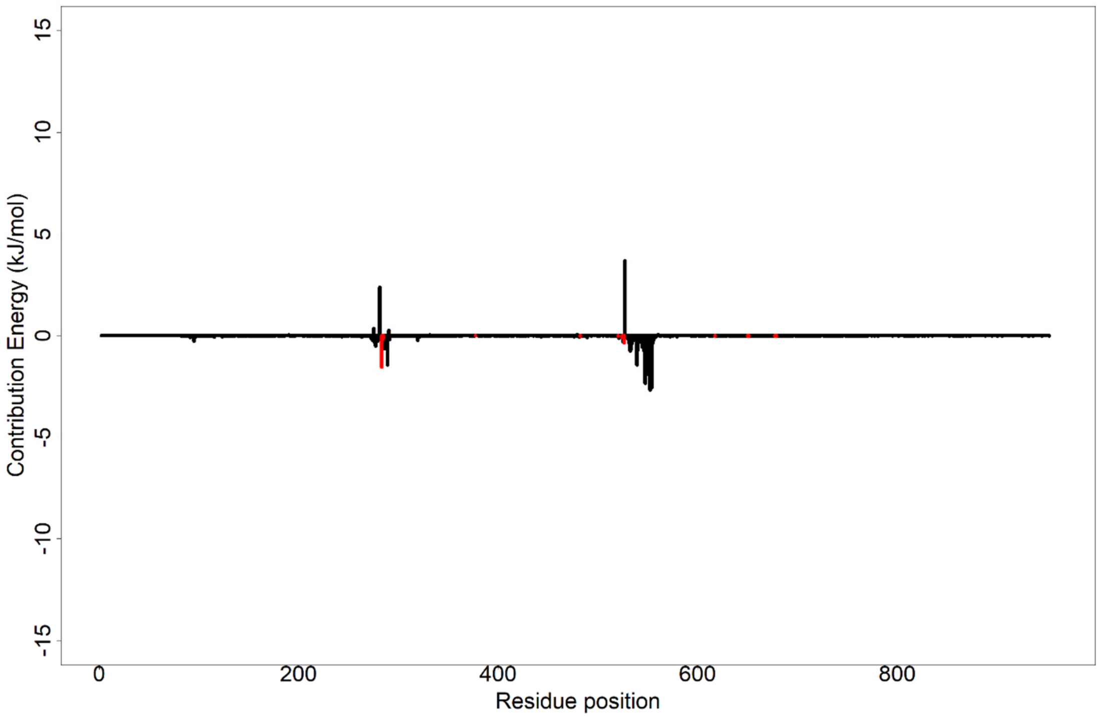

Figure S2. MM-PBSA plot of free binding energy contribution per residue decomposition of taraxasterol-alpha glucosidase complex.

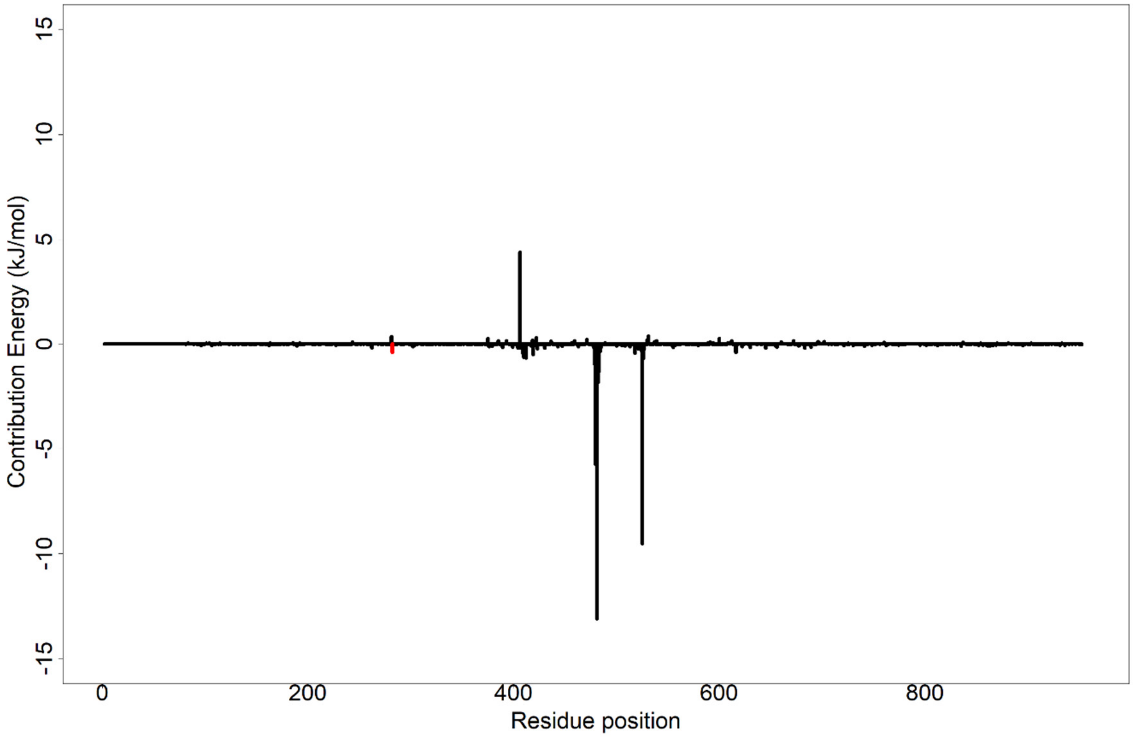

Figure S3. MM-PBSA plot of free binding energy contribution per residue decomposition of syriogenin-alpha glucosidase complex. *Hydrogen bonding residues labelled in red*

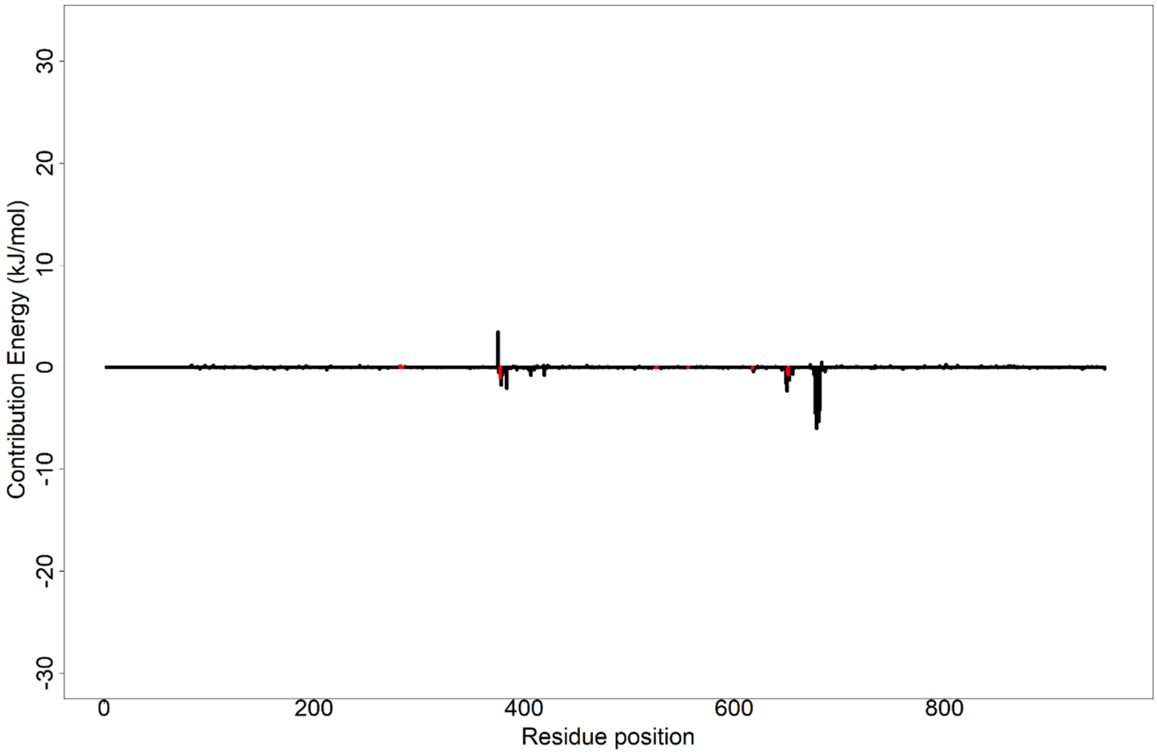

Figure S4. MM-PBSA plot of free binding energy contribution per residue of the complex of calotoxin with alpha-glucosidase. *Hydrogen bonding residues labelled in red*

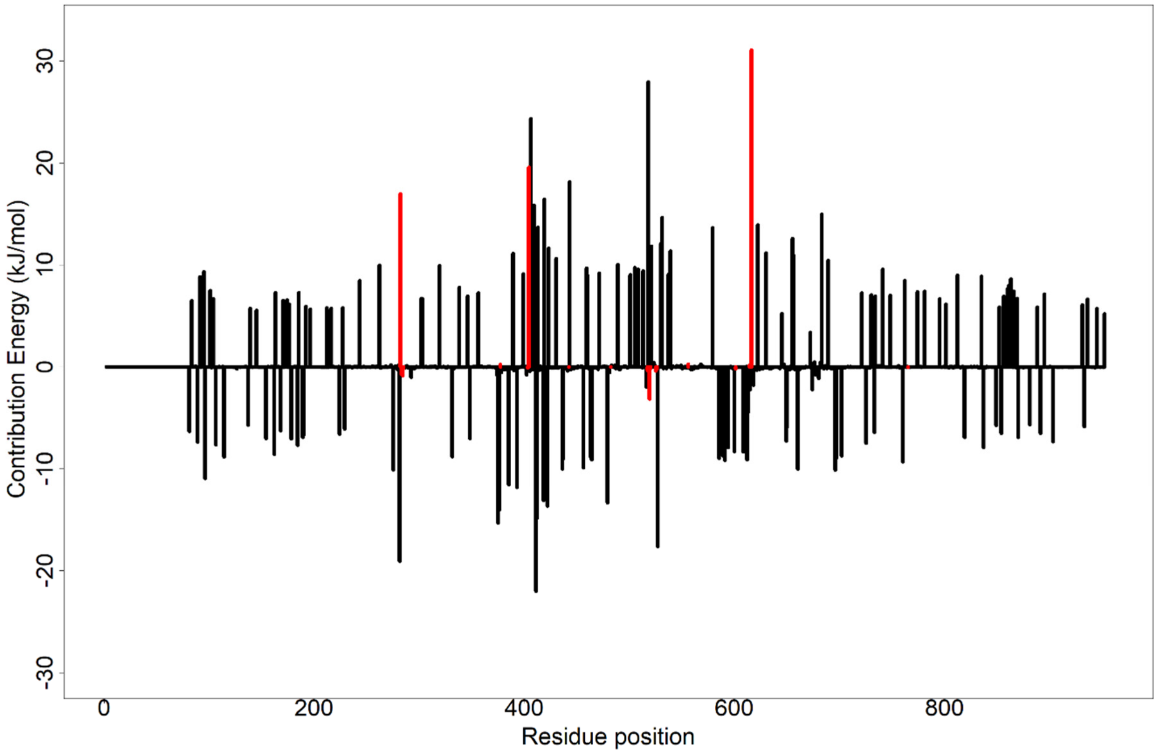

Figure S5. MM-PBSA plot of free binding energy contribution per residue of acarbose-alpha glucosidase complex. *Hydrogen bonding residues labelled in red.*

References

1. Yin, Z.; Zhang, W.; Feng, F.; Zhang, Y.; Kang, W. α-Glucosidase inhibitors isolated from medicinal plants. *Food Sci. Hum. Wellness* **2014**, *3*, 136–174. https://doi.org/10.1016/j.fshw.2014.11.003.
2. Dewi, R.T.; Maryani, F. Antioxidant and α-Glucosidase Inhibitory Compounds of Centella Asiatica. *Procedia Chem.* **2015**, *17*, 147–152. https://doi.org/10.1016/j.proche.2015.12.130.
3. Jia, Y.; Ma, Y.; Cheng, G.; Zhang, Y.; Cai, S. Comparative Study of Dietary Flavonoids with Different Structures as α-Glucosidase Inhibitors and Insulin Sensitizers. *J. Agric. Food Chem.* **2019**, *67*, 10521–10533. https://doi.org/10.1021/acs.jafc.9b04943.
4. William, J.; John, P.; Mumtaz, M.W.; Ch, A.R.; Adnan, A.; Mukhtar, H.; Sharif, S.; Raza, S.A.; Akhtar, M.T. Antioxidant activity, α-glucosidase inhibition and phytochemical profiling of Hyophorbe lagenicaulis leaf extracts. *PeerJ* **2019**, *7*, e7022. https://doi.org/10.7717/peerj.7022.
5. Chen, J.; Wu, S.; Zhang, Q.; Yin, Z.; Zhang, L. α-Glucosidase inhibitory effect of anthocyanins from Cinnamomum camphora fruit: Inhibition kinetics and mechanistic insights through in vitro and in silico studies. *Int. J. Biol. Macromol.* **2019**, *143*, 696–703. https://doi.org/10.1016/j.ijbiomac.2019.09.091.
6. Tabussum, A.; Riaz, N.; Saleem, M.; Ashraf, M.; Ahmad, M.; Alam, U.; Jabeen, B.; Malik, A.; Jabbar, A. α-Glucosidase in-hibitory constituents from Chrozophora plicata. *Phytochem. Lett.* **2013**, *6*, 614–619. https://doi.org/10.1016/j.phytol.2013.08.005.
7. Lestari, W.; Dewi, R.T.; Kardono, L.B.S.; Yanuar, A. Docking sulochrin and its derivative as α-glucosidase inhibitors of *Saccharomyces cerevisiae*. *Indones. J. Chem.* **2017**, *17*, 144–150.
8. Jhong, C.H.; Riyaphan, J.; Lin, S.H.; Chia, Y.C.; Weng, C.F. Screening alpha-glucosidase and alpha-amylase inhibitors from natural compounds by molecular docking in silico. *BioFactors* **2015**, *41*, 242–251. https://doi.org/10.1002/biof.1219.
9. Zahoor, M.; Bari, W.U.; Zeb, A.; Khan, I. Toxicological, anticholinesterase, antilipidemic, antidiabetic and antioxidant potentials of Grewia optiva Drummond ex Burret extracts. *J. Basic Clin. Physiol. Pharmacol.* **2019**, *31*, 20190220.
10. Kasturi, S.; Surarapu, S.; Uppalanchi, S.; Anireddy, J.S.; Dwivedi, S.; Anantaraju, H.S.; Perumal, Y.; Sigalapalli, D.K.; Babu, B.N.; Ethiraj, K.S. Synthesis and α-glucosidase inhibition activity of dihydroxy pyrrolidines. *Bioorganic Med. Chem. Lett.* **2017**, *27*, 2818–2823. https://doi.org/10.1016/j.bmcl.2017.04.078.

11. Zhu, J.; Zhang, B.; Tan, C.; Huang, Q. α-Glucosidase inhibitors: Consistency of: In silico docking data with in vitro inhibitory data and inhibitory effect prediction of quercetin derivatives. *Food Funct.* **2019**, *10*, 6312–6321. https://doi.org/10.1039/c9fo01333d.
